# Nervous NDRGs: the N-myc downstream–regulated gene family in the central and peripheral nervous system

**DOI:** 10.1007/s10048-019-00587-0

**Published:** 2019-09-04

**Authors:** Simone L. Schonkeren, Maartje Massen, Raisa van der Horst, Alexander Koch, Nathalie Vaes, Veerle Melotte

**Affiliations:** 1 Department of Pathology, GROW-School for Oncology and Developmental Biology, Maastricht University Medical Center, P.O. Box 616, 6200 MD Maastricht, The Netherlands; 2 Department of Clinical Genetics, Erasmus MC University Medical Center, Rotterdam, The Netherlands

**Keywords:** NDRG, Nervous system, Cancer, Charcot-Marie-Tooth disease, Dementia, Alzheimer’s disease

## Abstract

The N-Myc downstream-regulated gene (NDRG) family consists of four members (*NDRG1*, *NDRG2*, *NDRG3*, *NDRG4*) that are differentially expressed in various organs and function in important processes, like cell proliferation and differentiation. In the last couple of decades, interest in this family has risen due to its connection with several disorders of the nervous system including Charcot-Marie-Tooth disease and dementia, as well as nervous system cancers. By combining a literature review with in silico data analysis of publicly available datasets, such as the Mouse Brain Atlas, BrainSpan, the Genotype-Tissue Expression (GTEx) project, and Gene Expression Omnibus (GEO) datasets, this review summarizes the expression and functions of the NDRG family in the healthy and diseased nervous system. We here show that the NDRGs have a differential, relatively cell type–specific, expression pattern in the nervous system. Even though NDRGs share functionalities, like a role in vesicle trafficking, stress response, and neurite outgrowth, other functionalities seem to be unique to a specific member, e.g., the role of NDRG1 in myelination. Furthermore, mutations, phosphorylation, or changes in expression of NDRGs are related to nervous system diseases, including peripheral neuropathy and different forms of dementia. Moreover, NDRG1, NDRG2, and NDRG4 are all involved in cancers of the nervous system, such as glioma, neuroblastoma, or meningioma. All in all, our review elucidates that although the NDRGs belong to the same gene family and share some functional features, they should be considered unique in their expression patterns and functional importance for nervous system development and neuronal diseases.

## Introduction

The N-myc downstream–regulated gene (NDRG) family consists of four members: *NDRG1*, *NDRG2*, *NDRG3*, and *NDRG4*. The name of this family originates from the first gene discovered, *NDRG1*, as this gene can be repressed by the *c-myc* and *N-Myc* proto-oncogenes. However, even though each family member has been given a similar name, they are not all (in)directly regulated by either c- or N-myc [1, 2]. NDRG proteins share 57–65% amino acid identity and they all have an α/β hydrolase-fold region without hydrolytic catalytic activity [2, 3]. For a detailed review about the structure, origin, and function of the NDRG family, we refer to our previous review [2]. Briefly, all family members are functionally involved in cell proliferation, apoptosis, differentiation, development, and stress response, with NDRG1 and NDRG2 being extensively investigated in the context of cancer [4–6]. Generally, the NDRGs are considered to be tumor suppressor genes, by inhibiting proliferation and enhancing apoptosis through regulation of e.g. p53-, TGF-β-, and Wnt-signaling [7–9].

We previously identified *NDRG4* promoter methylation as a biomarker for the detection of colorectal cancer, and we observed that *NDRG4* is specifically expressed in neuronal cell bodies and nerve fibers in the intrinsic nervous system of the gut: the enteric nervous system (ENS) [10]. Next to *NDRG4*, *NDRG2*, and *NDRG3* are also expressed in enteric neural crest cells, the precursors of the ENS, during intestinal maturation, but their expression shifts towards other cell types in the adult gut [11]. Besides the role of NDRG4 in colorectal cancer, NDRG4 and the other NDRGs have also been described to be involved in nervous system cancers, like meningioma, neuroblastoma, and glioma. Moreover, all *NDRG* genes are expressed in nervous system structures and seem to be involved in the development and physiology of the nervous system [1]. All this addresses a potential importance for the NDRGs in the central and peripheral nervous system.

To learn more about the role of the NDRG family in the (patho)physiology of the nervous system, we performed an extensive literature search using Embase, Medline, Web of Science, and PubMed and validated these findings with in silico analyses using publicly available datasets, such as the Mouse Brain Atlas, the Genotype-Tissue Expression (GTEx) project (18/03/2019), and Gene Expression Omnibus (GEO) datasets (GSE9566 and GSE35366).

## Expression during development

The NDRG family members have different temporal expression patterns during the embryological phase and further development, suggesting that each member serves distinct purposes during development.

Detecting the mouse-analogues of *NDRG1*, *NDRG2*, and *NDRG3*, Okuda et al. found differences in mRNA expression patterns between the individual family members in the central nervous system (CNS). *NDRG3* was expressed relatively early in the embryological phase, around embryonic day 9.5 (E9.5), while *NDRG1* and *NDRG2* expressions only arose around E12.5 and E13.5. *NDRG1* was strongly expressed in the cerebral cortex, while *NDRG2* was specifically expressed around the ventricular zone in cerebrum and spinal cord [12]. Embryonic protein expression of NDRG2 was observed in the outer layer of the cortex, the choroid plexus, and the epidermis from E13.5 onward. In the adult mouse, this expression pattern changed to a more widespread distribution, particularly in the midbrain, cerebellum, and pons [13]. The expression of *NDRG2* was low, but widespread, in mouse and human fetal brain, and rose during postnatal development [14, 15]. *NDRG3* showed a broader expression pattern, both in cerebral cortex and spinal cord in mouse [12].

**Fig. 1:**
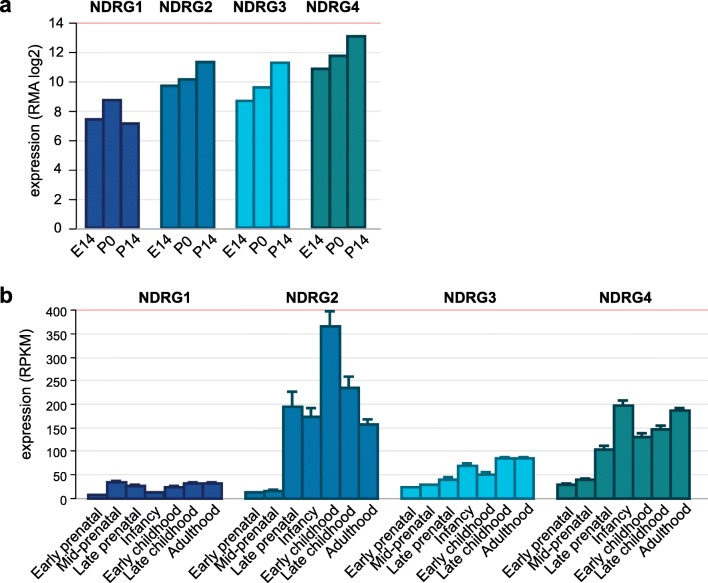
Temporal expression patterns of the NDRG family members during development. **a** Expression in developing wild-type mouse brain at three time points: embryonal day 14 (E14), postnatal day 0 (P0), and postnatal day 14 (P14). Expression values are Robust Multi-array Averages (RMA), corrected for background, log2 transformed, and quantile-normalized. Data were obtained from GEO (GSE35366) and analyzed using R (version 3.5.3). **b** Expression of the NDRGs in human brain samples throughout development, ranging from early prenatal stage to adulthood. Expression values are expressed as Reads per Kilobase Million (RPKM) ± SEM. Data were obtained from BrainSpan (http://www.brainspan.org/) and analyzed using the R2 Genomics Analysis and Visualization Platform (https://hgserver1.amc.nl/cgi-bin/r2/main.cgi).

The spatial expression pattern of the NDRG family in the CNS was also observed in *Xenopus tropicalis* [1]. *NDRG1* expression was mostly found in the forebrain, which later develops into the cerebrum, whereas *NDRG2*, *NDRG3*, and *NDRG4* expressions were found in the developing brain and spinal cord. However, temporal expression patterns differed from the mice studies, as *NDRG3* was expressed latest during development (gastrula stage 23) compared with the other family members (maternal expression in eggs) in *Xenopus tropicalis* [1]. In a time series of wild-type mouse brain samples from E14 to postnatal day 14 (P14), the expression of all *NDRG*s except for *NDRG1* rises during maturation (Fig. 1a) [16]. The same pattern can be seen in human brain samples from the BrainSpan project, ranging from the early prenatal period to adulthood [17, 18]. The expression of NDRG1 remains relatively low in the brain overall; NDRG2 expression initially increases, but declines after early childhood, while the expression of NDRG3 and NDRG4 increases over time (Fig. 1b).

Postnatally, *NDRG1* was expressed in the hippocampus from birth to P14 in rats, after which expression disappeared in the neurons of the hippocampus and arose in astrocytes in the caudate-putamen region in proximity to neurons. This could reflect the differentiation that hippocampal neurons and astrocytes undergo, as hippocampal neurons go through morphological and metabolic changes in the first two postnatal weeks when *NDRG1* is expressed, after which *NDRG1* expression arises in mature GFAP-positive astrocytes. This suggests that NDRG1 could play a role in the process of differentiation [19].

In zebrafish, *ndrg4* was exclusively expressed in the heart, CNS, and sensory system during embryonic development [20]. The ubiquitous expression of *ndrg4* in the CNS changed towards a more specific expression pattern in the cranial ganglia, hindbrain neurons, tegmentum, and cerebellum at 22–72 h post-fertilization [20]. Other studies investigating the expression profile of *NDRG4* in rats identified six different *NDRG4* transcript variants, three of which lack exon 18. The variants without exon 18 were detected in embryonic and early postnatal brains, the others in maturing and adult brains [21]. Using Western blotting, Nakada et al. detected a fourth NDRG4 protein isoform in rat brain, which also shows differential expression at various developmental time points [22]. Overall, *NDRG4* expression was found to be more abundant during the adult phase, when compared with the expression during the fetal phase in rats and humans [23].

The spatial expression patterns of the *NDRG* family members during embryology are summarized in Table 1. *NDRG1* expression is mainly restricted to the cerebrum during development, while *NDRG2*, *NDRG3* and *NDRG4* are also expressed in the spinal cord. Temporally, the expression of the NDRGs generally increases in the brain throughout development. Together, these expression data suggest that they might have a role in developmental or differentiation processes.

**Table 1:** Spatial expression of the NDRGs during development in several species

Gene name	Species	Expression area
*NDRG1*	*Xenopus tropicalis* [1]	Forebrain
	Rat [19]	Hippocampus
		Caudate-putamen
*NDRG2*	*Xenopus tropicalis* [1]	Brain
		Spinal cord
	Mouse [12, 13]	Ventricular zone cerebrum Spinal cord Choroid Plexus
*NDRG3*	*Xenopus tropicalis* [1]	Brain
		Spinal cord
	Mouse [12]	Cerebral cortex
		Spinal Cord
*NDRG4*	*Xenopus tropicalis* [1]	Brain
		Spinal cord
	Zebrafish [20]	Cranial ganglia
		Hindbrain
		Tegmentum
		Cerebellum

## Cellular expression

**Fig. 2:**
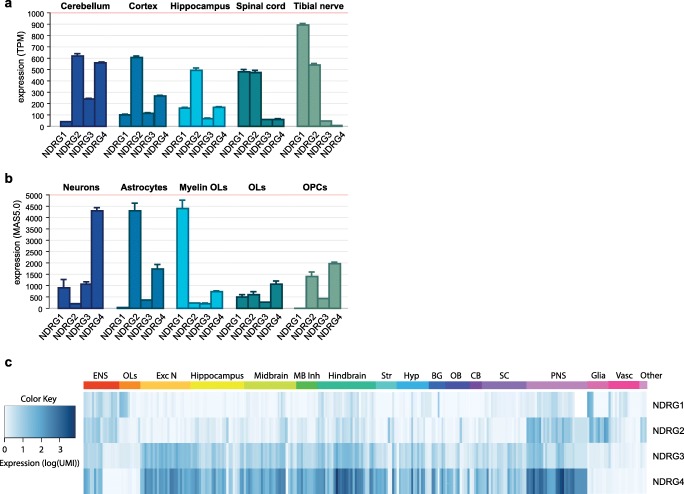
Spatial expression patterns of the NDRG genes in nervous system tissues and cell types. **a** RNA sequencing expression of the NDRG family members in the tibial nerve and different brain regions (human). The data used for the analyses were obtained from the GTEx project (v7) on 18/03/2019. TPM = Transcripts Per kilobase Million ± SEM. **b** Affymetrix GeneChip array expression analysis of the NDRG family members per cell type. Expression values are normalized using MAS5.0, data obtained from GEO (GSE9566). **c** Single-cell RNA sequencing expression of the NDRG family members in the nervous system. The data used for the analyses were obtained from http://mousebrain.org/genesearch.html on October 5, 2019. Abbreviations in **b**, **c** are as follows: Myelin OLs, myelinating oligodendrocytes; OLs, oligodendrocytes; OPCs, oligodendrocyte precursor cells; ENS, enteric nervous system; Exc N, excitatory neurons; MB Inh, midbrain inhibitory neurons; Str, striatum; Hyp, hypothalamus; BG, basal ganglia (thalamus and pallidum); OB, olfactory bulb; CB, cerebellum; SC, spinal cord; PNS, peripheral nervous system; Vasc, vascular cells

The expression patterns of the four NDRG family members do not only differ during embryologic development; their cellular distribution in the CNS and peripheral nervous system (PNS) during adulthood varies as well. Within the mouse brain, ***NDRG1*** is strongly expressed in oligodendrocytes and ependymal cells in the cerebrum (cortex), and weaker in Purkinje cells in the cerebellum (Fig. 2a) [24]. Oligodendrocyte localization was also observed in rats [25]. Moreover, NDRG1 is detected in the PNS, where it is mainly found in the cytoplasm of myelinating Schwann cells in rats, mice, and humans [25–27]. *NDRG1* is mainly expressed in the myelinating cell types which could be confirmed using the Mouse Brain Atlas and a GEO dataset (GSE9566) containing different types of CNS cells [28, 29]. *NDRG1* mRNA expression was found in myelinating and mature oligodendrocytes (Fig. 2b) and Schwann cells, but also in satellite glia, enteric glia, and nitrergic enteric neurons (Fig. 2c) [28].

**NDRG2** is strongly expressed in glia, as shown by its co-localization with glial fibrillary acidic protein (GFAP) in mice. NDRG2 has even been proposed as a specific marker for mature, non-reactive astrocytes, instead of or in addition to GFAP detection [30]. Mainly, astrocytes in the cerebrum and Bergmann glia in the cerebellum show cytoplasmic NDRG2 staining [24]. Shen et al. confirmed this and detected strongest NDRG2 levels in the midbrain and thalamus. The cerebral cortex, olfactory bulb, and hippocampus were also NDRG2-positive [31]. On RNA level, *NDRG2* is mostly expressed in astrocytes in the CNS (Fig. 2b) [29]. Using the Mouse Brain Atlas, we found *NDRG2* expression in fibrous astrocytes, GFAP-positive glia, and satellite glia. Interestingly, also sympathetic neurons of the PNS (noradrenergic and cholinergic) express *NDRG2* (Fig. 2c) [28]. In humans, the expression pattern of *NDRG2* is similar to mice, i.e., widespread throughout the CNS, including mRNA expression in cerebral cortex, striatum, cerebellum, brain stem, and spinal cord (Fig. 2a) [14]. NDRG2 protein is specifically localized in the majority of astrocytes, as shown by co-localization with GFAP and S100 calcium-binding protein B (S100β) and lack of co-localization with the neuronal marker microtubule-associated protein 2 (MAP2) and neuron-specific enolase (NSE) in the brain [30]. On the contrary, NDRG2 was not only detected in GFAP-positive astrocytes but also in neuronal nuclei (NeuN)–positive neurons in human fetal brain at gestational week 28 [15]. Thus, NDRG2 is predominantly, but not exclusively, expressed in astrocytes in the nervous system.

Although NDRG1 and NDRG2 share a cytoplasmic localization in CNS and PNS cells, **NDRG3** is mainly localized in the nucleus. Its expression is strongest in neurons, as confirmed by a double staining with NeuN in mice. Cerebellar Purkinje cells and granule cells were also NDRG3-positive, although the latter to a lesser extent [24]. Overall, *NDRG3* mRNA expression is strongest in the cortex, mostly in excitatory neurons, and in hindbrain, mostly in excitatory, cholinergic, and serotonergic neurons (Fig. 2c) [28].

We and others observed that **NDRG4** has a similar expression pattern as NDRG3 (e.g., cerebral neurons, cerebellar Purkinje cells), but is specific to the cytoplasm [10, 24]. NDRG4 always co-localizes with the neuronal marker HuC/D and NeuN, but never with the glial marker GFAP, indicating its specific neuronal expression [10], which is confirmed by the highest expression of *NDRG4* mRNA in neurons in the brain (Fig. 2b). NDRG4 is almost exclusively expressed in nervous system structures throughout the body, including the CNS, PNS, and ENS. Within the CNS, NDRG4 mRNA and protein expression was observed in the cerebrum, namely the cerebral cortex, mesencephalon, pons and medulla oblongata, the cerebellum (Purkinje cells), and the spinal cord (Fig. 2a) [10, 23]. In mice, *NDRG4* mRNA expression is highest in the peripheral sensory neurons in the dorsal root ganglion, sympathetic neurons, and excitatory and cholinergic hindbrain neurons in the CNS (Fig. 2c) [28].

Overall, the NDRG family members seem to have a distinct cellular organization in the nervous system. NDRG1 is mainly constricted to myelinating cell types (e.g., oligodendrocytes and Schwann cells); NDRG2 mostly localizes in astrocytes in the CNS, while NDRG3 and NDRG4 are mainly detected in neurons. Although NDRG1, NDRG2, and NDRG4 share a cytoplasmic expression pattern, NDRG3 is specifically located in the nucleus. These variable expression patterns might explain some of the functional differences between the family members, which will be discussed below.

## Functions

Although limited functional research within the PNS and CNS has been done on the ***NDRG1*** gene, it has been linked to nerve myelination, stress response, lipid biosynthesis and metabolism, exocytosis, and differentiation [32]. As described above, *NDRG1* is exclusively expressed by myelinating cell types (e.g., Schwann cells within the PNS and oligodendrocytes within the CNS). In addition, mutations in *NDRG1* are known to cause a peripheral neuropathy related to demyelination, namely Charcot-Marie-Tooth disease type 4D (CMT4D), which we will further discuss in detail in the next section [25, 33]. Rosalind et al. investigated the function of NDRG1 in relation to myelination using both hypomorphic *NDRG1* knockout (KO) mice and mice with a complete deficiency of NDRG1 (the stretcher mouse (str.)) [34]. In both models, initial myelination was normal, but axonal damage arose after 3–5 weeks, which resulted in decreased nerve conduction velocity. Interestingly, the str. model had a markedly more severe phenotype, suggesting that even a very low expression of NDRG1 can partly rescue the phenotype.

Differential expression analysis between healthy peripheral nerves and *NDRG1*-deficient nerves revealed that NDRG1 is involved in lipid trafficking. Moreover, NDRG1 is a partner protein of Prenylated Rab Acceptor 1 (PRA1), required for vesicle trafficking from the Golgi complex [34]. This indicates that NDRG1 could be one of the CMT-associated proteins involved in endosomal transport mechanisms, like SH3TC2 in CMT4C [35].

 NDRG1 also has an important role in lipid metabolism, which is a crucial process for the formation of myelin [36]. Pietiäinen et al. first studied the influence of NDRG1 on lipid transport in epithelial cells [37]. They found a decrease in low-density lipoprotein (LDL) uptake upon silencing of *NDRG1* using small interfering RNAs (siRNA). This was caused by a reduced abundance of LDL-receptors on the plasma membrane. The same effect of NDRG1 silencing on LDL trafficking was found in mouse oligodendrocytes, which elucidates a role for NDRG1 in normal lipid and cholesterol trafficking. In addition, the differentiation factor oligodendrocyte lineage transcription factor 2 (Olig2) was downregulated in *NDRG1* deficient oligodendrocytes, suggesting that NDRG1 is also involved in oligodendrocyte differentiation.

Moreover, NDRG1 has a role in stress conditions. NDRG1 is the substrate of serum/glucocorticoid-regulated kinase 1 (SGK1), which is activated by plasma corticosterone. Expression and activation of SGK1 increase specifically in oligodendrocytes in response to increased plasma corticosterone levels and causes an increase in NDRG1 phosphorylation. This leads to different downstream effects, including increased expression levels of the main adhesion molecules, e.g., N-cadherin, α-catenin, and β-catenin, and altered morphology of oligodendrocytes. Repeated exposure to stress led to excess arborization of oligodendrocyte processes in mice, and addition of the synthetic glucocorticoid dexamethasone to cultured oligodendrocytes caused an increase in their cell size. The same effect was established by overexpressing active SGK1 and phosphorylated NDRG1, implicating that the morphological changes are a result of the stress-induced SGK1-NDRG1 pathway [38, 39]. It is evident that NDRG1 is an important protein in myelinating cell types, with functions ranging from maintenance of myelination and lipid transport to oligodendrocyte differentiation and stress-response.

As mentioned above, ***NDRG2*** is predominantly expressed in astrocytes in the CNS. Gene silencing of *NDRG2* in cultured mouse astrocytes increased proliferation while *NDRG2* overexpression inhibited proliferation, suggesting that NDRG2 suppresses proliferation in astrocytes [40]. Furthermore, NDRG2 might influence astrocyte morphology, as *NDRG2* silencing caused the formation of shorter processes and reduced F-actin content [40]. The antiproliferative function of NDRG2 was also observed in C6-originated astrocytes (differentiated C6 glioma cells). Li et al. investigated the involvement of NDRG2 during p53-induced apoptosis seen in cerebral ischemia/reperfusion injury. Silencing of *NDRG2* in cultured astrocytes alleviated the apoptotic effect of oxygen-glucose deprivation while overexpression of *NDRG2* augmented apoptosis via changes in the Bax/Bcl-2 ratio (apoptosis-promoting and apoptosis-suppressing mitochondrial membrane proteins). Furthermore, oxygen-glucose deprivation resulted in a p53-dependent upregulation of NDRG2 and translocation of NDRG2 to the nucleus [41]. Upregulation of NDRG2 was also seen in rat brain directly after cerebral ischemia, after which NDRG2 levels declined again [42].

Another study revealed a role for NDRG2 in gliotransmission, as an indirect modulator of kainate receptor subunit expression. Like NDRG1, NDRG2 is a substrate of SGK1. NDRG2 phosphorylation was found to suppress the SGK1-induced increased membrane expression of the glutamate receptor subunit GluK2 in rat primary astrocytes. SGK1 is upregulated during stress and NDRG2 might attenuate the stress response by preventing an excessive incorporation of GluK2 in the membrane [43]. This could be a potential mechanism as to why the expression of the glutamate transporters glutamate aspartate transporter (*GLAST*) and glutamate transporter 1 (*GLT-1*) were increased by deletion of *NDRG2*. An alternative explanation would be that the increased expression of glutamate transporters was caused by increased activation of Akt-signaling, as *NDRG2* silencing resulted in increased levels of p-Akt [44].

Finally, the role of NDRG2 on the formation of neuronal structures has been investigated in NGF-treated PC12 cells. Takahashi et al. observed an increase in *NDRG2* mRNA expression during neurite outgrowth. Moreover, NDRG2 protein localized specifically to the cell membrane and growth cones, and overexpression of *NDRG2* in these cells caused neurite elongation. This suggests that NDRG2 could play a role in the formation of (neuronal) processes [45].

The function of **NDRG3** within the nervous system has barely been investigated. *NDRG3* appears to be a hypoxia-responsive gene that is upregulated during cerebral ischemia in rats. Resembling *NDRG2*, its expression rises during the first phases of ischemic injury/hypoxia and diminishes after the injury [46]. This could correspond to the upregulated expression of other neuroprotective genes during hypoxia, such as VIP and PACAP [47]. NDRG3 might thus function as a neuroprotective protein, although other functions of NDRG3 in the nervous system remain to be elucidated.

Only recently, the function of **NDRG4** in the nervous system has been investigated showing that ndrg4 plays a prominent role in signal transduction via myelinated axons in zebrafish [48]. *Ndrg4*-deficiency impaired the physiological function of the nodes of Ranvier, which cluster sodium channels to ensure fast transduction. Sodium channel clustering was nearly absent in *ndrg4* mutants, leading to impaired signal transduction, even though myelination of the axon was intact. QPCR, Western blot, and immunohistochemistry revealed that ndrg4 regulates some key genes of the vesicle docking pathway, e.g., synaptosomal-associated protein 25 (Snap25). Defective vesicle docking due to *ndrg4* knockdown was at least partially responsible for the impaired sodium channel clustering. Ndrg4 thus functions in vesicle release and plays a fundamental role in the development and organization of myelination in the peripheral nervous system in zebrafish [48].

Yamamoto et al. used an *NDRG4*KO mouse model to investigate its effects on the nervous system. The cortex of *NDRG4*KO mice contained lower levels of brain-derived neurotrophic factor (BDNF), which led to impaired spatial learning and memory in the Morris water maze [49]. The association between NDRG4 and BDNF was further investigated in rat brain, where ischemic injury initially upregulated NDRG4 expression, but ultimately caused decreased levels of NDRG4 and a concomitant decrease in BDNF levels. Upregulation of NDRG4 through injection with an adenoviral vector rescued BDNF levels [50]. This indicates that NDRG4 is necessary for the maintenance of BDNF levels.

NDRG4 is also involved in p53-mediated apoptosis in ischemic injury in rats. After an initial increase of NDRG4, decreased levels of NDRG4 were observed, resembling the expression of *NDRG2* and *NDRG3* during cerebral ischemia. Upregulation of NDRG4 after ischemic injury could suppress neuronal apoptosis by decreasing Bax expression in mitochondrial fractions and by inhibiting the direct interaction with p53, as shown by co-immunoprecipitation [51]. NDRG4 thus seems to be involved in regulation of apoptosis in the brain.

Similar to the role of NDRG2, NDRG4 is also upregulated during neuronal differentiation of PC12 cells. Silencing of *NDRG4* resulted in inhibition of neurite outgrowth through the suppression of activator protein 1 (AP-1) transcription factor activation [52, 53]. NDRG4 is likely important for neuronal differentiation, possibly by increasing phosphorylation of ERK1/2, downstream targets in the MAPK/ERK pathway, the major pathway that induces neuronal differentiation [54]. Thus, NDRG2 and NDRG4 seem to be positive regulators of neurite outgrowth and PC12 neuronal differentiation.

**Fig. 3:**
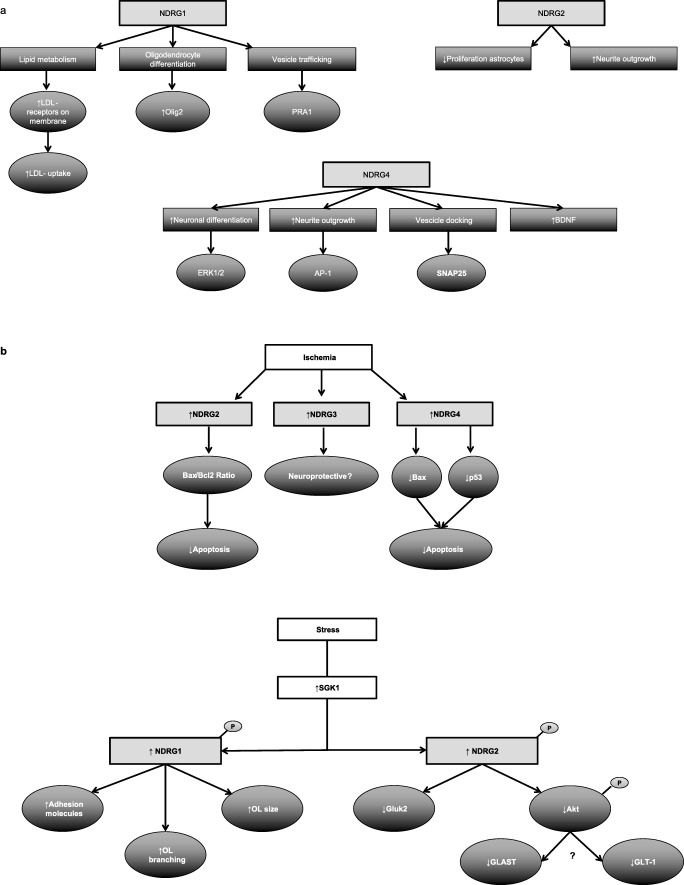
Functional pathways influenced by NDRG1, NDRG2, NDRG3, and NDRG4. **a** Distinct functional pathways for NDRG1, NDRG2, and NDRG4. **b** Shared functional pathways induced by ischemia and stress involving more than one NDRG family member. LDL, low-density lipoprotein; Olig2, oligodendrocyte lineage transcription factor 2; PRA1, Prenylated Rab Acceptor 1; BDNF, brain-derived neurotrophic factor; AP-1, activator protein 1; Snap25, synaptosomal-associated protein 25; SGK1, serum/glucocorticoid-regulated kinase 1; OL, oligodendrocyte; GLAST, glutamate transporter glutamate aspartate transporter; GLT-1, glutamate transporter 1

Even though the *NDRG* family members share roughly 60% amino acid identity, their cellular expression in the nervous system is distinct and so are many of their functions. As NDRG1 is specifically expressed in myelinating cell types, it is not surprising that it plays a role in myelination, possibly through regulation of lipid metabolism. NDRG2 is mostly investigated for its effect on proliferation and apoptosis, and appears to repress proliferation in astrocytes. NDRG3 appears to play a role in ischemia where it could act as a neuroprotective gene. NDRG4 seems to be involved in vesicle trafficking and apoptosis after ischemic injury. In addition, some functions are shared, like the role of NDRG1 and NDRG2 in stress response and the involvement of NDRG2 and NDRG4 in neurite outgrowth. NDRG2, NDRG3, and NDRG4 also seem to be involved in brain ischemia. A schematic overview of the pathways influenced by NDRG1, NDRG2, and NDRG4 are shown in Fig. 3a. Shared functional pathways involving more than one NDRG family member are shown in Fig. 3b.

## Association with pathologic conditions: nervous system malignancy

### Charcot-Marie-Tooth disease type 4D

Charcot-Marie-Tooth disease type 4D (CMT4D), also referred to as hereditary motor and sensory neuropathy-Lom (HMSNL), is a peripheral neuropathy that primarily occurs in the Gypsy community. The autosomal recessive disease usually results from a homozygous R148X mutation in ***NDRG1***, but can also be caused by other mutations in *NDRG1*, such as IVS8-1G>A, or frameshift mutations [55]. Although the NDRG1 mutation R148X generally only affects the PNS, two cases in a non-Gypsy family showed white matter abnormalities in the CNS [56]. The disease is clinically characterized by muscle weakness, sensory loss, and neural deafness. Pathologic alterations include Schwann-cell dysfunction with “onion bulbs”, leading to hypomyelination and demyelination/remyelination [27]. In a rat model investigating de- and remyelination, the sciatic nerves that were transected (to prevent regeneration) show decreased *NDRG1* mRNA levels, while sciatic nerves that were crushed (where regeneration was possible) have reduced *NDRG1* mRNA expression shortly after the injury, which returns to normal after remyelination is complete [25]. This indicates that the expression pattern of *NDRG1* correlates to the myelin content.

Owing to the fact that NDRG1 is specifically expressed by myelinating glia such as oligodendrocytes and Schwann cells, it can be expected that the expression of mutant NDRG1 protein contributes to the pathogenesis of such a demyelinating neuropathy [25]. The other family members do not seem to play a role in demyelinating neuropathies, as they are not, or to a far lesser extent, expressed in myelinating cell types.

### Alzheimer’s disease

Alzheimer’s disease (AD) is a form of dementia characterized by two main pathological hallmarks, namely accumulation of intracellular neurofibrillary tangles and senile plaques [57]. Interestingly, ***NDRG2*** was found to be one of the most pronounced upregulated genes in hippocampi of AD patients compared with healthy controls. Mitchelmore et al. investigated the expression of NDRG2 in human hippocampal biopsies from eight patients with confirmed late onset AD and five controls. Both RNA and protein expression of NDRG2 were found to be elevated twofold in AD-affected brains compared with healthy control brains. Expression of NDRG2 was localized to cortical pyramidal neurons, dystrophic neurons, and senile plaques, which are all affected by AD [14]. Similarly, the expression level of NDRG2 was also increased in a genetic rat model of AD [58]. One of the proposed mechanisms how NDRG2 can affect AD pathogenesis is that neuronal cell death can be induced by NDRG2-phosphorylation through death-associated protein kinase 1 (DAPK1) activation. DAPK1 is activated by senile plaques and ceramide and has a higher expression in human AD brain samples, as well as NDRG2-phosphorylation levels [59]. The effect of NDRG2-phosphorylation on neuronal proliferation has not been investigated elsewhere, so further research is necessary to clarify the exact role of NDRG2 on neuronal degeneration. Another possibility is that NDRG2 influences amyloid precursor protein (APP) metabolism or amyloid β-plaque formation. NDRG2 expression was found to be higher in aged rats and rats with injected Aβ_1-42_, a model for AD. *NDRG2* silencing led to a decrease in Aβ_1-42_ (the predominant form of amyloid β found in AD) in neuroblastoma cells, and overexpression to an increase in Aβ_1-42_, implicating NDRG2 in the formation of senile plaques. Moreover, tau-phosphorylation, the primary mechanism of neurofibrillary tangle-formation, was upregulated by NDRG2 [60]. This suggests that NDRG2 is important in both main pathological alterations in AD.

In contrast, **NDRG3** and **NDRG4** were found to be downregulated in AD patients’ brains [61, 62], which could be related to the decrease in BDNF levels that was seen in e.g. *NDRG4*KO mice [49]. Despite these findings, no further studies have been done to investigate the role of these family members in AD.

### Frontotemporal lobar degeneration

Frontotemporal lobar degeneration (FTLD) is a neurodegenerative disease that mostly leads to abnormal behavior, personality changes, and language dysfunction. Using a phosphoproteomic analysis on postmortem human brain tissue from FTLD and age-matched controls, Herskowitz et al. found that **NDRG2** and GFAP phosphorylation were increased compared with controls [63]. Considering that NDRG2 and GFAP are proposed as markers for fibrous astrocytes, these findings implicate that fibrous astrocytes could be of importance in FTLD. However, as cause-consequence is difficult to assess in these studies, it is unclear whether NDRG2-phosphorylation actively participates in FTLD pathology.

### Multiple sclerosis

Multiple sclerosis is a demyelinating disease, disrupting the communication between parts of the nervous system.*NDRG2* was investigated in the context of neuroinflammation, using an *NDRG2*KO mouse in an experimental model of multiple sclerosis (experimental autoimmune encephalomyelitis (EAE)). Deletion of *NDRG2* reduced clinical symptoms of EAE. Although it had minor effects on inflammation, *NDRG2-*deficiency caused neurodegeneration in the acute phase of EAE and also affected oligodendrocytes in the chronic phase. The protecting effect of NDRG2 can be explained by its role in glutamate receptor restoration. In fact, the reduced glutamate receptor expression and concomitant glutamate toxicity in EAE can be counteracted by the NDRG2-induced upregulation of glutamate aspartate transporter (GLAST) and glutamate transporter 1 (GLT-1). Due to this, demyelination and neurodegeneration, the characteristic features in multiple sclerosis, were significantly reduced [44].

### Meningioma

Meningiomas, which are neoplasms originating from arachnoid cells, represent a significant proportion of all primary nervous system neoplasms. Although meningiomas are mostly benign, around 15% of meningiomas are aggressive and exhibit the potential to invade the normal brain tissue and to frequently and destructively recur [64]. The WHO has defined three grades, namely benign (grade I), atypical (grade II), and anaplastic/malignant (grade III) meningiomas, representing about 80%, 15–20%, and 1–3%, respectively [65].

**NDRG2** expression has been linked with tumor grade and tumor recurrence. Using qPCR, Skiriute et al. investigated *NDRG2* expression in 35 patients with primary and recurrent meningiomas. Recurrent meningiomas displayed a statistically significant reduction in *NDRG2* mRNA levels, when compared with primary meningiomas. Furthermore, *NDRG2* gene expression was found to be significantly decreased by 3.7-fold in atypical (grade II) meningiomas when compared with benign (grade I) meningiomas [65]. Although the patient cohort in this study was relatively small (*n* = 35), similar statistically significant results were obtained by Lusis et al. (*n* = 49) who identified *NDRG2* as a tumor suppressor gene. Loss of NDRG2 mRNA and protein levels was found in anaplastic (grade III) meningiomas and in a clinically aggressive subset of atypical (grade II) meningiomas, most likely caused by *NDRG2* CpG promoter methylation [66].

Although these studies found a strong relationship between loss of *NDRG2* expression and tumor grade/recurrence, a larger study by Ongaratti et al. (*n* = 60) did not observe this effect using immunohistochemistry [67]. The lack of reproducibility could have been caused by the small number of meningiomas with invasive and aggressive characteristics in this study (*n* = 12 for tumor grades II and III combined), or by the fact that the two studies used different antibodies for immunohistochemistry. It is therefore still likely that loss of *NDRG2* expression is related to tumor aggressiveness, although it is difficult to draw any conclusions regarding the correlation with protein expression. Functionally, it has been described that NDRG2 has an antiproliferative effect, so loss of NDRG2 would be beneficial for tumor growth. Combining these findings, we hypothesize that *NDRG2* should be considered a tumor suppressor gene in meningiomas.

Kotipatruni et al. obtained data suggesting that **NDRG4** has a proto-oncogenic role in aggressive meningioma. Using in vitro models, they showed that *NDRG4*-silencing induced apoptosis and reduced the invasive potential of the meningioma cell lines IOMM-Lee and CH-157 MN. Thus, it seems that NDRG4 is necessary for meningioma cells to survive [64, 68]. No other (clinical) studies are available that investigate NDRG4 in the context of meningioma, so it is unclear whether NDRG4 is indeed involved in meningioma pathology in patients.

### Neuroblastoma

Neuroblastoma is typically a childhood cancer, accounting for approximately 10% of all pediatric cancers. The tumor is derived from neural crest cells of the sympathetic nervous system, but the exact pathological mechanism remains unknown [69]. ***NDRG1*** likely serves as a tumor suppressor in neuroblastoma development. mRNA and protein expression were investigated in 48 tissue specimens from neuroblastoma patients, and low NDRG1 expression was significantly associated with prognostic factors such as primary tumor size, MYCN amplification, and poor prognosis [70]. *NDRG1*-overexpression in the c-Myc-overexpressing neuroblastoma cell line SK-N-MC caused reduced cell size and reduced colony formation, confirming the tumor-suppressive role of NDRG1 [71]. NDRG1 expression in neuroblastoma cells can also be induced by the transcription factor forkhead box D3 (FOXD3), which has a lower expression in neuroblastoma tissues and cell lines (SH-SY5Y and SK-N-SH) [69].

In patients with neuroblastoma (*n* = 42), high **NDRG2** expression resulted in significantly longer survival. NDRG2 expression was lower in neuroblastoma tissue and cells than normal dorsal ganglia and was significantly associated with the tumor suppressor intelectin 1 (ITLN1) [72]. To determine the functional role of NDRG2 in neuroblastoma, overexpression of *NDRG2* was induced in the neuroblastoma cell lines SK-N-SH and SH-SY5Y, which resulted in an increased expression of the tumor suppressor gene protocadherin 17 and differentiation-related genes such as Rsu1 and Smurf1, and the decreased expression of proliferation-related genes such as CYR61. NDRG2 overexpression could thereby reduce cell proliferation compared with control cells [73].

### Glioma

Malignant gliomas are the most common malignant primary brain tumor and also the most aggressive tumor type of the nervous system [74]. Compared with the abovementioned nervous system malignancies, NDRG expression profiles in glioblastoma have been extensively researched. Gliomas are either astrocytic, oligodendrocytic, or a mix of these cell types from origin, and they can be classified from WHO grades I–IV according to malignancy. Different designations are used for gliomas in the literature, including astrocytoma (grades I–II), anaplastic astrocytoma (grade III), and glioblastoma (grade IV) [75]. In this article, we will use the WHO grading system to indicate tumor malignancy and to compare different studies.

The **NDRG1** expression pattern was investigated in tissue sections of grade II glioma (*n* = 40) where moderate-to-high NDRG1 protein expression was found as a prognostic factor for reduced risk of glioma progression and progression-free survival. However, overall survival of glioblastoma patients was not significantly affected by NDRG1 expression levels [76]. A larger study investigating different glioma grades (*n* = 168) found reduced NDRG1 expression in gliomas, which was negatively correlated to glioma grade. This study reported a significantly lower overall survival in patients with low NDRG1 expression, independent of other prognostic indicators (e.g. tumor grade) [77]. Cell line experiments in glioma cells showed that NDRG1 inhibits cell proliferation and invasion and induced apoptosis. Additionally, tumorigenicity of subcutaneously injected NDRG1-overexpressing glioma cells was reduced in vivo [78], concluding that NDRG1 likely has a tumor-suppressive function in glioma.

Contrary to these findings, Said et al. observed higher NDRG1 mRNA and protein expression levels in grade IV glioma (*n* = 15) compared with grade II glioma (*n* = 15) and suggested that this is caused by the hypoxic state of the tumor, as NDRG1 is a downstream target of hypoxia-inducible factor 1 (HIF-1). However, this study did not link NDRG1 expression to survival rate or prognosis [79]. Weiler et al. found that hypoxia and radiotherapy induced NDRG1 expression in glioma cells, leading to a poor response to alkylating chemotherapy [80]. Blaes et al. confirmed this by observing that post-surgically treated patients with genotoxic-induced NDRG1 expression had reduced overall survival, while patients that had not been post-surgically treated had an increased overall survival related to high NDRG1 expression [76]. Collectively, these data suggest that NDRG1 might have properties of a tumor suppressor gene in glioblastoma, but that upregulation by genotoxic treatment will impair the response to chemotherapy and lead to reduced overall survival.

**NDRG2** has also been found as a candidate tumor suppressor gene in glioma as NDRG2 expression was markedly reduced in grade IV glioma tissues (*n* = 27). Moreover, overexpression of NDRG2 in U373 and U138 (glioblastoma cell lines) led to reduced cell proliferation [81]. Another study reported NDRG2 expression as an independent prognostic factor for overall survival in glioma patients. NDRG2 expression was investigated in grade I–IV glioma tissue samples (*n* = 316) and was found to be decreased in more aggressive glioma grades compared with healthy controls [82]. In addition, NDRG2 promotor methylation was found to be tumor-specific and associated with shorter survival in patients who survived less than 24 months (4.6 months, methylated; 7.8 months, non-methylated), but was not associated with overall survival (*n* = 137) [83]. Three other studies confirmed the finding that NDRG2 expression is lower in gliomas due to NDRG2 promoter methylation. Tepel and colleagues [84] observed decreased NDRG2 protein and mRNA expression in grade IV gliomas compared with grade II and grade III gliomas (*n* = 67) and observed hypermethylation in 62% of grade IV gliomas (*n* = 34). Zhou et al. [85] observed lower *NDRG2* mRNA expression in glioma tissue (*n* = 53) compared with adjacent healthy tissue (*n* = 26). In addition, the methylation rate of the NDRG2 promoter was with 46.3% in glioma tissue significantly different from the 18.2% in normal tissue. Skiriute et al. analyzed grade I–IV gliomas (*n* = 137) and confirmed that gene methylation frequency increased in higher grade gliomas and mRNA and protein expression decreased. However, this study found no correlation between NDRG2 expression and promoter methylation. Clinical significance was also investigated and revealed a significantly longer survival time for patients with unmethylated NDRG2 status, high mRNA expression of NDRG2, and high protein NDRG2 expression, although they could not be used as separate prognostic factors [86].

In contrast to NDRG2, the studies regarding expression of **NDRG4** in glioma show conflicting outcomes. One study described increased NDRG4 expression, while others report downregulation of NDRG4 in glioma [87–90]. Ding et al. observed significantly decreased NDRG4 protein and mRNA expression in glioma tissue (*n* = 49) compared with normal tissue (*n* = 10), which was also confirmed using The Cancer Genome Atlas (TCGA; *n* = 410) data [87], and by Kolodziej et al. [89]. In addition, reduced NDRG4 protein expression was found as a predictor for poor prognosis and reduced overall survival in both low- and high-grade gliomas (*n* = 128) [90].

Contrary to these findings, Schilling et al. detected higher NDRG4 expression in glioma grade IV tissue (*n* = 6) compared with healthy brain tissue (*n* = 2). However, the number of patients investigated in this study is too low to draw any conclusions. Follow-up analysis focused on in vitro experiments, concluding that NDRG4 is important for cell cycle progression and cell viability [88]. Kolodziej et al. reported a small increase in NDRG4 protein levels in glioma tissue as observed with immunohistochemistry, mainly located in glia, but this was not validated by any other methods. The authors themselves propose that this finding is due to non-specific staining [89]. NDRG4 thus seems to be a tumor suppressor gene in glioma. However, considering that NDRG4 is exclusively expressed in neurons, it is debatable whether NDRG4 would be of major influence in a glia-originated cancer.

Dysfunction of the NDRG family members can clearly lead to pathological alterations, as is best described for NDRG1 in CMT4D. However, neurodegenerative diseases like Alzheimer’s disease and frontotemporal lobar degeneration might also be influenced by NDRG1 or NDRG2, although these mechanisms remain to be elucidated. NDRG3 has not been described to be involved in any nervous system cancer type. However, NDRG3 has been described in several other cancer types, such as prostate cancer, non-small cell lung cancer, hepatocellular carcinoma, breast cancer, and laryngeal squamous cell carcinoma [91–93]. Additional research is required to identify a potential role for NDRG3 within the nervous system and nervous system malignancies. In the context of nervous system cancers, *NDRG1*, *NDRG2*, and *NDRG4* have been described to be tumor suppressor genes, although some results are not consistent throughout different studies. These discrepancies could be related to the use of different antibodies for protein detection, small sample sizes, or different interventions in patients, such as genotoxic treatments. All in all, we can conclude that *NDRG* family members are important for normal functioning of the nervous system and that alterations in gene expression can lead to disease conditions.

## Conclusion

The NDRG family is largely represented in the nervous system of multiple species. Expression already arises during embryonic development and remains present during adulthood. The NDRG family has been linked to differentiation and proliferation processes, which might explain their role in development. In spite of their involvement in similar processes, it is important to note the diversity of the NDRGs regarding their expression pattern and functional roles in the nervous system. However, the NDRGs show specific expression patterns in distinct cell types and they also have diverse functions in the nervous system. Pathological alterations of the NDRGs, like mutations and altered phosphorylation or expression levels, can cause a variety of diseases, including neurodegenerative diseases and nervous system cancers. Although some NDRGs are specifically involved in a disease without contribution of the other family members, a common feature of the NDRG family seems their involvement in various cancer types. Although most research currently focuses on NDRGs in cancer, it is important to keep in mind that the function of the NDRGs is not restricted to cancer hallmarks, such as proliferation and apoptosis, but ranges from lipid and vesicle trafficking to sodium channel clustering important for normal neuronal/cellular functioning. Furthermore, all NDRGs should be considered as individual genes, as their expression patterns as well as their functions are mostly distinct. Nonetheless, more research has to be done before we can elucidate the exact role of each NDRG family member in the nervous system.
